# Exosomal miR-125b Exerts Anti-Metastatic Properties and Predicts Early Metastasis of Hepatocellular Carcinoma

**DOI:** 10.3389/fonc.2021.637247

**Published:** 2021-07-27

**Authors:** Hye Seon Kim, Jin Seoub Kim, Na Ri Park, Heechul Nam, Pil Soo Sung, Si Hyun Bae, Jong Young Choi, Seung Kew Yoon, Wonhee Hur, Jeong Won Jang

**Affiliations:** ^1^Department of Biomedicine & Health Sciences, College of Medicine, The Catholic University of Korea, Seoul, South Korea; ^2^The Catholic University Liver Research Center, College of Medicine, The Catholic University of Korea, Seoul, South Korea; ^3^Department of Internal Medicine, College of Medicine, The Catholic University of Korea, Seoul, South Korea; ^4^Division of Chronic Viral Disease, Center for Emerging Virus Research, National Institute of Infectious Disease, National Institute of Health, Chungbuk, South Korea

**Keywords:** hepatocellular carcinoma, exosome, metastasis, epithelial – mesenchymal – transition, biomarker, miR-125b

## Abstract

**Background & Aims:**

Cancer metastasis is responsible for the majority of cancer-related deaths. Exosomal miRNAs have emerged as promising biomarkers for cancer, serving as signaling molecules that can regulate tumor growth and metastasis. This study examined circulating exosomal miRNAs that could predict hepatocellular carcinoma (HCC) metastasis.

**Methods:**

Exosomal miRNA was measured by quantitative real-time PCR (qRT-PCR) in a large set of patients (*n* = 284). To investigate the role of exosomal miRNA in HCC, we performed a series of *in vitro* tests, such as exosome labeling, qRT-PCR, reverse transcription PCR, wound healing assay, transwell assay, and Western blot assay.

**Results:**

Exosomal miR-125b was drastically downregulated in HCC patients with metastasis than in those without metastasis. *In vitro*, we observed the uptake of miR-125b by exosome in recipient cells. Exosome-mediated miR-125b significantly inhibited migration and invasion abilities and downregulated the mRNA expressions of MMP-2, MMP-9, and MMP-14 in recipient cells *via* intercellular communication. Further investigation revealed that miR-125b suppressed SMAD2 protein expression in recipient cells by binding to its 3′ untranslated regions. Exosome-mediated miR-125b transfer also disrupted TGF-β1–induced epithelial–mesenchymal transition and TGF-β1/SMAD signaling pathway in recipient cells by leading to a decrease of SMAD2 protein expression. Moreover, exosomal miR-125b was downregulated after metastasis compared with that at baseline in patients with serial measurements before and after metastasis.

**Conclusions:**

The results imply that exosome-mediated miR-125b exerts anti-metastatic properties in HCC. These findings highlight that circulating exosomal miR-125b might represent a reliable biomarker with diagnostic and therapeutic implications for extrahepatic metastasis from HCC.

## Introduction

Hepatocellular carcinoma (HCC) is a lethal cancer with the third highest mortality in the world. One of the most adverse prognostic events of HCC is vascular invasion contributing to treatment resistance and extrahepatic metastasis. Cancer metastasis refers to the dissemination of malignant cells to distant sites through blood vessels and is responsible for the majority of cancer-related deaths (1). To ensure patient survival and the potential for a cure for patients presenting with metastasis, it is essential to predict or diagnose cancer metastasis in its early stages. Considering that metastasis is spread by various biological signals in the blood, biomarkers for metastasis are expected to be present in the blood. Unfortunately, there is still a lack of valid and reliable biomarkers for early detection of metastasis from HCC.

Exosomes, 30 to 150 nm nanosized extracellular vesicles that are secreted from a wide variety of cells into biological fluids, have received extensive attention because they act as cell-to-cell communication mediators by horizontally transferring their cargos, including nucleic acids, proteins, and lipids (2). MicroRNAs (miRNAs) are short non-coding RNA molecules that regulate gene expressions post-transcriptionally and cellular functions epigenetically by directly binding to 3′ untranslated regions (UTRs) of target mRNAs (3). Given that specific miRNAs are useful in clinical applications for disease (4), exosome-encapsulated miRNAs that overcome tumor heterogeneity are clinically relevant and deserve further investigation (5, 6). Accumulating evidences have shown that tumor-derived exosomes promoted the spread of metastasis by transferring various signals through the blood (7). Moreover, the exosome-mediated transfer of specific miRNAs contributes to behaviors of metastatic capacity *via* paracrine and endocrine signaling (5, 8, 9). Thus, exosomal miRNAs could open an innovative window as promising biomarkers of metastasis in the future. Nevertheless, circulating exosomal miRNAs that can predict extrahepatic metastasis have not been extensively studied in HCC.

The aim of this study was to explore exosomal miRNAs that could predict extrahepatic metastasis in patients with HCC. By analyzing miRNA profiles, we identified the potential role of circulating exosomal miR-125b as a biomarker for early detection of metastasis from HCC. Subsequent studies on mechanisms underlying exosome–target cell interactions indicated that the transfer of miR-125b by exosomes suppressed migration and invasion abilities of recipient cells by attenuating epithelial to mesenchymal transition (EMT) *via* inhibition of TGF-β1/SMAD signaling. Moreover, novel biomarker functions of exosomal miR-125b were confirmed in a large set of patients with HCC.

## Materials and Methods

### miRNA Microarray

To analyze microarray, serum samples were collected from patients with or without HCC. Microarray analysis was performed by GenoCheck (Ansan, Korea). In brief, total RNA was extracted from serum and labeled with alkaline phosphatase. Hybridization was then performed using an Agilent hybridization system on Agilent Mouse miRNA v17.0 array to conduct DNA chip assay. Raw data were analyzed using GeneSpring GX v11.5.1 to evaluate miRNA expressions.

### Patient Samples

This study examined serum samples from 239 HCC patients and 45 non-HCC patients at Seoul St. Mary’s Hospital, Catholic University of Korea (Seoul, South Korea) between June 2007 and January 2019. Among these, serial measurements for exosomal miRNA were performed for nine HCC patients who had serial samples available before and after metastasis. The diagnosis of HCC was based on histological evidence, α-fetoprotein levels, or typical radiological findings according to the KNCC guideline (10). Metastasis was diagnosed based on pathology, bone scan, computed tomography, or magnetic resonance imaging. Based on tumor extent (11), patients diagnosed as HCC were categorized into the following three groups: 1) “under Milan group,” a single tumor < 5 cm or multiple tumors (number ≤ 3, each < 3 cm in diameter) without metastasis; 2) “over Milan group,” HCC exceeding Milan criteria but without metastasis; and 3) “metastasis group,” HCC exhibiting extrahepatic metastasis. This study was approved by the ethics committee of The Catholic University of Korea. Informed written consent was obtained from all patients (IRB approval number KC17TESI0664).

### Exosome Isolation and Characterization

Exosomes were isolated from sera and cell culture-conditioned media (CM) using an ExoQuick™ (System Biosciences, Palo Alto, CA, USA) and a total exosome isolation kit (TEI; Invitrogen, Carlsbad, CA, USA), respectively. In brief, serum was centrifuged at 3000*g* for 15 min at 4°C to remove cellular debris. Exosomes were then isolated from sera according to the manufacturer’s instructions. To isolate exosomes from CM, cells were washed with PBS when reaching 80% confluence and incubated with serum-free media (SFM) for 48 h. CM was collected and then ultrafiltered with Amicon Ultra Centrifugal Filters (Millipore, Bedford, USA). Exosomes were subsequently isolated from CM according to the manufacturer’s instructions. For exosome characterization, the exosome pellet was resuspended in PBS. Exosomes were visualized by transmission electron microscopy (TEM). Size distribution and quantification of exosomes were determined using nanoparticle tracking analysis (NTA). Exosomal markers were detected by Western blot assay.

### miRNA Transfection Into Cells and Exosomes

HCC cells were transfected with hsa-miR-125b-5p mimic (miR-125b; Genolution Pharmaceuticals, Seoul, Korea) and negative control mimic (miR-NC; Genolution Pharmaceuticals) using Lipofectamine 2000 (Invitrogen) according to the manufacturer’s instructions. The final concentration of miRNA mimics used in this study was 50 nM. Exosomes were also loaded with miRNA mimics based on a previously reported method (12). In brief, Huh7 cell-derived exosomes (Huh7-exo) were loaded with miR-125b (Exo-125b) and miR-NC (Exo-NC) using Lipofectamine 2000 (Invitrogen). These miRNA-loaded exosomes were purified using TEI (Invitrogen) to remove any un-transfection mixture. Transfection or loading efficiency was analyzed by qRT-PCR.

### Co-Culture Experiment

Recipient cells (SK-HEP1 and SNU449 cells) were seeded into six-well plates. After reaching 80% confluency, these cells were treated with Exo-125b or Exo-NC suspended in SFM for 24 h.

### Transfer of Exosomes and Exosomal miRNA in Cell-to-Cell Communication

To assess the transfer of miRNA by exosomes, Huh7-exo were loaded with Cy3-labeled miR-125 (Genepharma, Shanghai, China) or miR-NC as described above. These miRNA-loaded exosomes were labeled with a PKH67 green fluorescent cell linker for general cell membrane labeling (Sigma-Aldrich, St. Louis, MO, USA) according to the manufacturer’s instructions. Following purification of exosomes using TEI, recipient cells were co-cultured with exosomes for 24 h. Images were then taken with a confocal microscopy (LSM800, Carl Zeiss, Germany).

### Cell Migration and Invasion Assay

Wound healing and transwell assays were performed to assess cell migration and invasion abilities. In brief, following transfection in six-well plates, cell monolayers were wounded with a sterile yellow tip. Cells were then washed and replaced with completed media supplemented with 10% FBS. Images were taken 24 and 48 h later using an optical microscopy. Transwell assays were conducted using corning insert and Biocoat matrigel invasion chamber (Corning Inc, Corning, NY, USA) according to the manufacturer’s instructions. In brief, cells were trypsinized following transfection and resuspended in SFM. The cell suspension was seeded into each upper chamber after rehydration in SFM for 2 h. The lower chamber was added with 10% FBS-containing media and incubated for 48 h at 37°C in 95% air and 5% CO_2_. Migrated and invaded cells were stained with Diff-Quick (Sysmex, Japan) and counted.

### Matrix Metalloproteinase (MMP) Expression

The mRNA expression of MMPs was examined by reverse transcription polymerase chain reaction (RT-PCR). Total RNA was extracted from recipient HCC cells using Qiazol reagent (Qiagen, Germany). cDNA was synthesized using high-capacity cDNA reverse transcription kit (Applied Biosystems, Foster City, CA,USA). The primer pairs used for the detection of MMP-2, MMP-9, and MMP-14 are shown in **Table S1**. The cDNA was amplified with 10-µM primers using Maxime PCR PreMix Kit (Intron Biotechnology, Seoul, Korea). GAPDH was used as an endogenous control.

### EMT Cell Model

To induce EMT, after miRNA-loaded exosomes co-culture, Huh7 cells were treated with 5 ng/ml transforming growth factor beta-1 (TGF-β1; R&D system, Minneapolis, MN, USA) for 48 h. Following incubation, RNA and protein were extracted from cells for further experiments.

### Statistical Analysis

All statistical analyses were performed using GraphPad Prism (GraphPad Software, La Jolla, CA, USA) and SPSS 20.0 software (IBM, Armonk, NY, USA). Data were presented as mean ± standard error of the mean (SEM) or median. Comparisons between groups were appropriately performed using Student’s *t*-test, Mann-Whitney U test, or Wilcoxon signed-rank test. Survival analysis was analyzed with Kaplan-Meier method and log-rank test. Statistical significance was denoted as *p < 0.05; **p < 0.01; ***p < 0.001.

### Additional Information

Additional experimental methods, including cell culture, Western blot assay, reverse transcription, and quantitative real-time polymerase chain reaction (qRT-PCR), are provided in supporting data.

## Results

### Circulating miRNAs Screening in HCC Patients

To identify circulating miRNAs for HCC tumorigenicity, miRNA microarray was performed using sera of HCC and non-HCC cirrhotic patients. A total of 10 miRNAs were selected based on the following criteria: fold change ≥ 1.5 and *p* value < 0.05 (**Figure S1A**). Among them, two promising miRNAs of our interest and three additional miRNAs found by searching PubMed were analyzed by qRT-PCR. As a result, upregulation of miR-125b and miR-100 and downregulation of miR-3180, miR-130a, and miR-320a were found to be associated with HCC (**Figure S1B**). Of these, considering that miR-125b and miR-100 act as tumor suppressor miRNAs in HCC (13, 14), they were further analyzed in our study. To determine expressions of candidate miRNAs in exosomes, we first attempted to isolate exosomes from patients’ sera. Isolated vesicles were characterized by TEM for visual confirmation (**Figure S2A**). NTA was also performed to determine size distribution and concentration (**Figure S2B**). The presence of HSP70 and CD63 commonly used as exosomal markers was detected in vesicles (**Figure S2C**), confirming the successful isolation of exosomes. In serum exosomes of HCC patients, two miRNAs (miR-125b and miR-100) were confirmed by qRT-PCR. Of these, circulating exosomal miR-125b expression was significantly downregulated in the sera of HCC patients with metastasis compared to those without metastasis (*p* = 0.030; **Figure S2D**). Based on the abovementioned results, we hypothesized that exosomal miR-125b could regulate extrahepatic metastasis from HCC.

### Exosomal miR-125b Can Transfer to Recipient Cells

**Figure 1:**
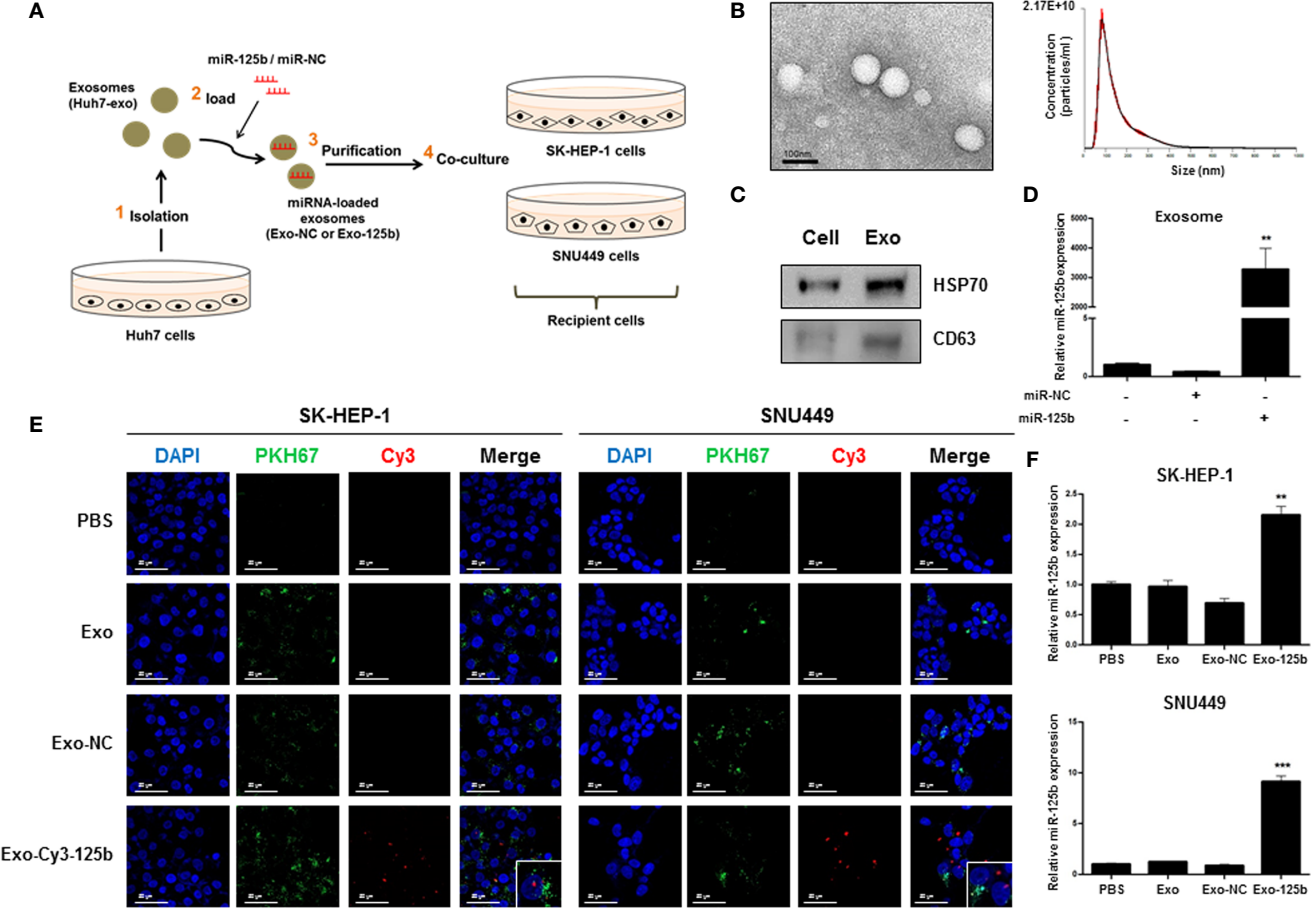
Exosomes mediate the transfer of miR-125b into recipient cells. **(A)** Schematic diagram showing the co-culture experimental procedure. In step 1, exosomes were isolated from Huh7-CM. In step 2, isolated exosomes were loaded with miRNA mimics. In step 3, miRNA-loaded exosomes were purified by TEI. In step 4, purified exosomes were co-cultured with recipient cells. **(B)** Exosomes were characterized by TEM and NTA. Scale bar, 100 nm. **(C)** Detection of exosomal markers in cell lysate and exosomes. **(D)** Upregulation of relative miR-125b expression in Exo-125b compared with that in Exo-NC. **(E)** Confocal microscopy images of recipient cells treated with Exo-Cy3-125b (Cy3-labeled miR-125b-loaded exosomes) or Exo-NC. Original magnification, ×400 or ×800. Scale bar, 50 µm. Red: Cy3-labeled miR-125b; green: exosome; DAPI: nuclei. **(F)** Relative miR-125b expression determined by qRT-PCR after recipient cells were treated with Exo-125b or Exo-NC. Data are presented as mean ± SEM. **p < 0.01; ***p < 0.001.

To test the above hypothesis, we performed co-culture experiments using exosomes (**Figure 1A**). We employed cells with high-metastatic potential (SK-HEP-1 and SNU449 cells) as recipient cells and low-metastatic cells (Huh7 cells) as donor cells to investigate the role of exosomal miR-125b in metastasis (15–17). Exosomes were isolated from Huh7 cells and characterized as shown in **Figures 1B, C**. Overexpression of miR-125b in Exo-125b compared with that in Exo-NC was confirmed (**Figure 1D**). As indicated in **Figures 1E, F**, exosomal miR-125b internalization into recipient cells was observed by confocal microscopy and confirmed by qRT-PCR. Altogether, these results indicate that miR-125b can be loaded into Huh7-exo and taken up into recipient cells *via* exosome transfer.

### Exosomal miR-125b Inhibits Migration and Invasion Capacities of Recipient Cells

**Figure 2:**
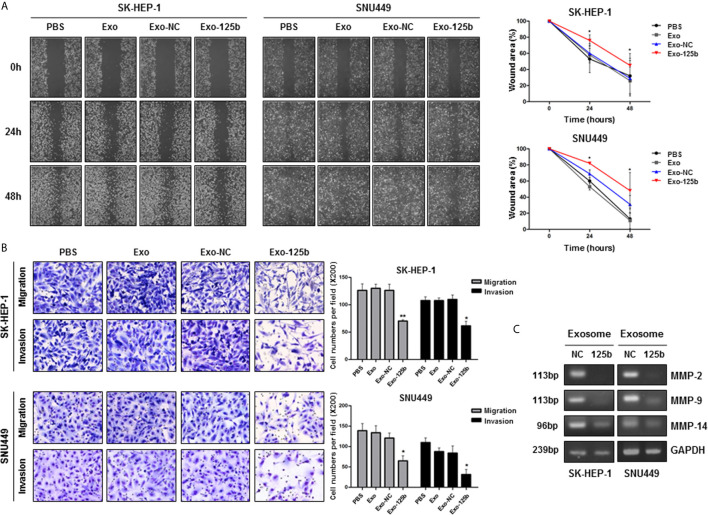
Exosomal miR-125b inhibits migration and invasion abilities of HCC cells. Cell migration and invasion abilities were assessed by wound healing and transwell assays. **(A)** Percent (%) of wound area was determined as the ratio of average wound closure at a given time points (24, 48 hours) relative to the initial wound closure. **(B)** Numbers of migration and invasion cells were counted in indicated groups. **(C)** RT-PCR results displayed reduced mRNA expression levels of MMP-2, MMP-9, and MMP-14 in recipient HCC cells treated with Exo-125b compared to those in cells treated with Exo-NC. Data are presented as mean ± SEM. *p < 0.05; **p < 0.01 *vs.* Exo-NC.

Cellular miR-125b was reported to inhibit migration and invasion abilities of HCC cells (13). Overexpression of miR-125b significantly impaired wound healing capacity compared with that of miR-NC (**Figures S3A, B**). However, Huh7 cells known to have low-metastatic capacity did not show noticeable difference in wound healing capacity between miR-125b and miR-NC mimics. Next, we examined effects of exosome-mediated miR-125b on recipient cells. Similar to the results observed in cells, wound healing capacity was suppressed in recipient cells treated with Exo-125b (**Figure 2A**). Moreover, transwell assay showed that the number of migration and invasion cells was significantly decreased in recipient cells treated with Exo-125b (**Figure 2B**). MMPs are known to promote cancer migration and invasion (18). Expression of all the MMPs in different type was found to be strongly repressed in recipient cells treated with Exo-125b as compared with those treated with Exo-NC (**Figure 2C**). Taken together, these results suggest that exosomal miR-125b exerts tumor suppressive function by inhibiting metastatic ability of recipient cells *via* cell-to-cell transfer.

### SMAD2 Is a Direct Target of Exosomal miR-125b in Recipient Cells

**Figure 3:**
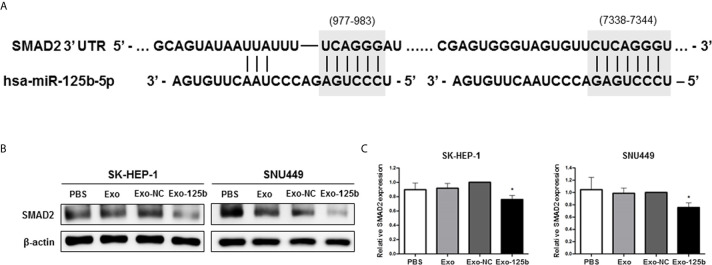
SMAD2 is a direct target of exosomal miR-125b in recipient HCC cells. **(A)** Two potential seed regions (gray) indicate the binding site of miR-125b in SMAD2 3′ UTR. **(B)** Western blot assay showing decreased protein expression of SMAD2 after treatment with Exo-125b compared to that after treatment with Exo-NC. **(C)** Statistical analysis of SMAD2 protein expression in recipient HCC cells. Data are presented as mean ± SEM. *p < 0.05 *vs*. Exo-NC.

miRNAs are involved in various cellular activities by repressing protein expressions of target genes (3). Thus, to identify target genes of miR-125b for anti-metastatic properties, we used open-source bioinformatics algorithms, including TargetScan, miRWalk, miRDB, TargetRank, and Exiqon. Candidate target genes were screened by Western blot assay (**Figure S4**). Among various candidates, SMAD2 protein expression was most significantly suppressed in Huh7 cells transfected with miR-125b mimic. With functional annotation analysis by Database for Annotation, Visualization, and Integrated Discovery, the target genes of miR-125b were most strongly associated with the TGF-β signaling pathway (*p* = 0.004; **Table S2**). Two seed regions of miR-125b and SMAD2 were predicted with TargetScan and well matched as shown in **Figure 3A**. This result is consistent with previous reports showing that SMAD2 is a target of miR-125b in HCC and that it is a strongly related to cancer metastasis (19, 20). To determine whether miR-125b regulates protein expression of SMAD2 in recipient cells by intercellular communication, recipient cells were overexpressed by treatment with Exo-125b. As a result, SMAD2 protein expression was found to be significantly decreased in recipient cells after treatment with Exo-125b (**Figures 3B, C**). These results indicate that exosomal miR-125b suppresses post-transcriptional SMAD2 protein expression in recipient cells through intercellular communication.

### Exosomal miR-125b Attenuates EMT Induced by TGF-β1 and Blocks TGF-β1/SMAD Pathway

**Figure 4:**
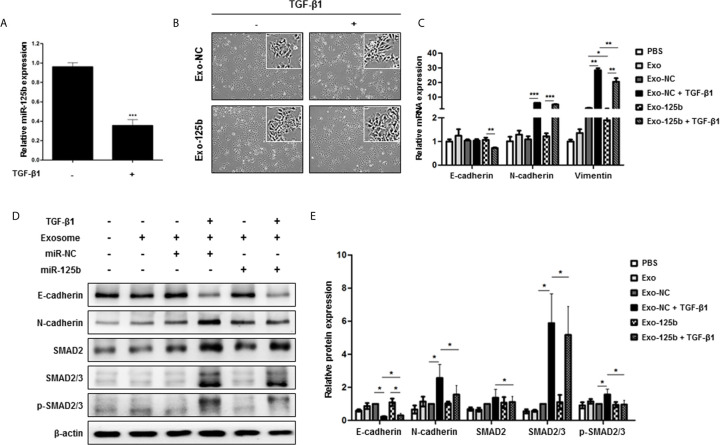
Exosomal miR-125b blocks TGF-β1-induced EMT and TGF-β1/SMAD pathway signaling in recipient HCC cells. Low metastatic Huh7 cells were induced to show EMT by TGF-β1. **(A)** Relative miR-125b expression in Huh7 cells following treatment with TGF-β1. **(B)** Changes of morphology observed by optical microscopy after EMT induction by TGF-β1. **(C)** qRT-PCR results of mRNA expression levels of EMT marker genes in cells. **(D)** Protein expression levels of TGF-β1-EMT pathway genes based on Western blot assay. **(E)** Statistical analysis of protein expression levels in cells. Relative expression levels were normalized β-actin. Data are presented as mean ± SEM. *p < 0.05; **p < 0.01; ***p < 0.001.

TGF-β plays a pivotal role in EMT and SMAD2 is a key regulator of TGF-β signaling pathway (21). Thus, we further explored whether miR-125b could interfere with TGF-β signaling pathway by suppressing SMAD2. When Huh7 cells were treated with TGF-β1, miR-125b expression was significantly downregulated (**Figure 4A**). To elucidate the relevance of EMT and miR-125b, low metastatic Huh7 cells were used as recipient cells to induce EMT. After TGF-β1 treatment, the Exo-125b group only showed changes slightly with a spindle-shaped morphology while the Exo-NC group clearly displaying spindle-shaped cells (**Figure 4B**). As indicated in **Figure 4C**, mRNA expression level of an epithelial marker (E-cadherin) was decreased in response to TGF-β1, whereas expression levels of mesenchymal markers (N-cadherin and Vimentin) were significantly increased. Overexpression of vimentin mRNA by TGF-β1 was significantly reduced after Exo-125b treatment. Next, we examined changes in protein levels of EMT markers in response to TGF-β1. Consistently, E-cadherin was downregulated, whereas N-cadherin was upregulated in Huh7 cells (**Figures 4D, E**). Although either Exo-NC or Exo-125b treatment alone resulted in no significant change in protein expressions of target genes or EMT markers, combined treatment with Exo-125b and TGF-β1 resulted in significant upregulation of E-cadherin protein expression and significant downregulation of N-cadherin, SMAD2, SMAD2/3, and p-SMAD2/3, suggesting a drastic suppression of metastatic potential by transfer of exosomal miR-125b in EMT-promoting cells. Collectively, these findings demonstrate that exosomal miR-125b can block EMT and TGF-β1/SMAD pathway by repressing protein expression of SMAD2.

### Exosomal miR-125b Is Downregulated in the Sera of HCC Patients With Metastasis

**Figure 5:**
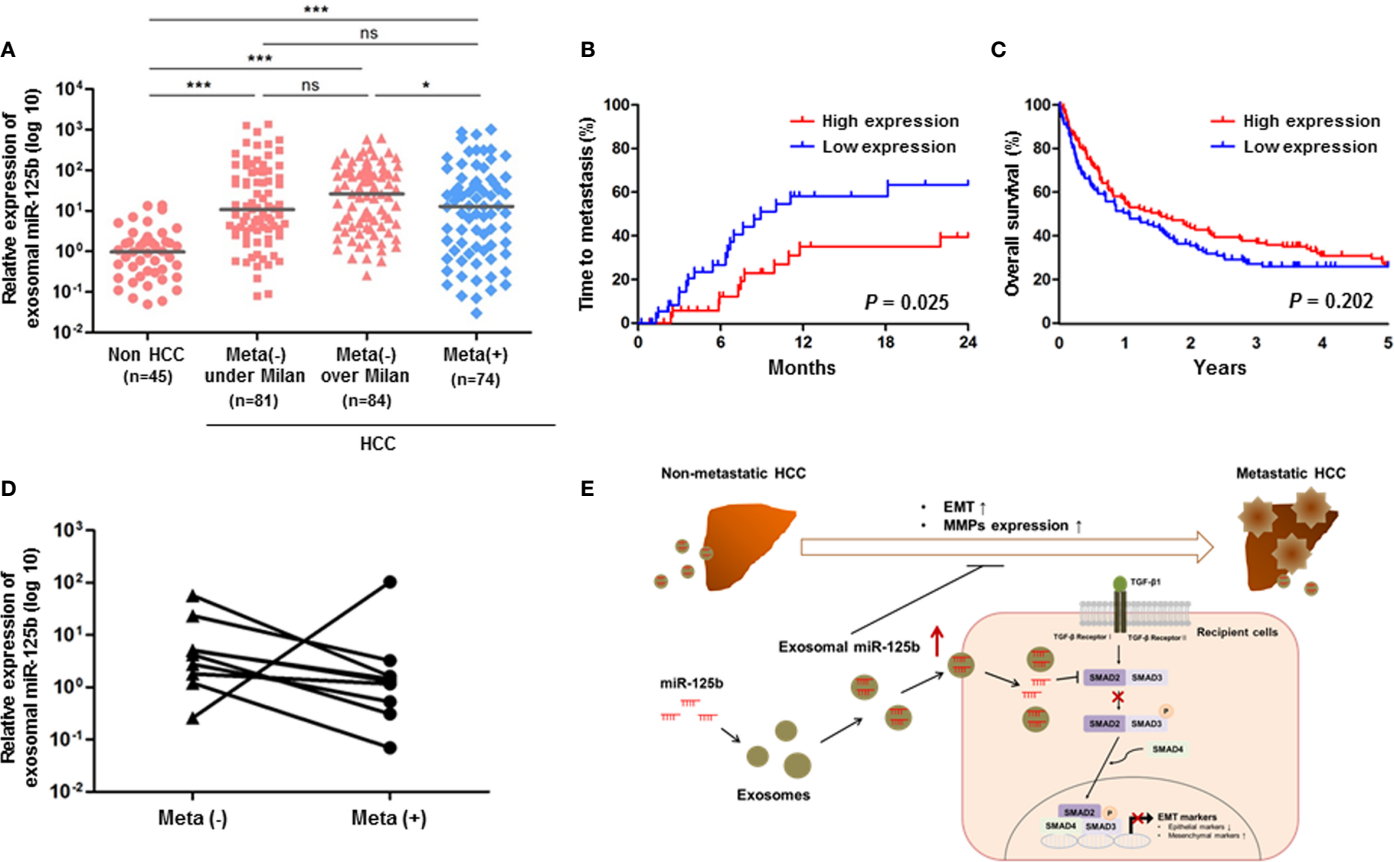
Circulating exosomal miR-125b is a strongly predictive biomarker for extrahepatic metastasis from HCC. **(A)** Relative expression of exosomal miR-125b in sera of HCC patients (*n* = 239) and non-HCC patients (*n* = 45). **(B, C)** Kaplan-Meier survival analysis of time to metastasis for meta(-) over Milan patients group and 5 years overall survival. **(D)** Comparison of exosomal miR-125b expression in HCC patients with serial samples available before and after metastasis (*n* = 9). **(E)** A paradigm of exosomal miR-125b regulating extrahepatic metastasis in HCC. Data are presented as median. Mann-Whitney U test or Wilcoxon signed-rank test was used for data analysis. ns, not significant; *p < 0.05; ***p < 0.001.

Based on our *in vitro* results of exosome-mediated miR-125b, we evaluated whether its anti-metastatic properties could serve as a biomarker for early detection of metastasis or therapeutic implications in patients with HCC. For this purpose, we tested exosomal miR-125b expression in sera of 284 patients with available sera samples. As a result, exosomal miR-125b expression was correlated with patient outcomes, showing increasing trends with tumor stage progression. However, these expression levels were significantly decreased with metastasis (**Figure 5A**). Survival analysis was examined based on circulating exosomal miR-125b expression profiles. The low exosomal miR-125b expression group had higher rates of extrahepatic metastasis (*p* = 0.025), as well as trends for worse overall survival (*p* = 0.202) than the high expression group (**Figures 5B, C**). Furthermore, when analyzing patients with serial samples available before and after metastasis, we found that exosomal miR-125b expression was significantly downregulated after metastasis in all patients but one (**Figure 5D**). Overall, these data indicate that exosomal miR-125b is a strong predictor of early extrahepatic metastasis in HCC patients. Clinical characteristics of patients are provided in **Table 1**.

**Table 1:** Baseline characteristics of patients with hepatocellular carcinoma.

Characteristics	(n = 239), n (%)
Sex	
Male	185 (77.4)
Female	54 (22.6)
Age (years)	60 ± 11.6
Causes	
Viral	201 (84.1)
Non-viral	38 (15.9)
AST (U/L)	57 (14-799)
ALT (U/L)	54 ± 47.7
TB (mg/dL)	0.9 (0.2-18.2)
AFP level (ng/ml)	101.6 (0.9-448240)
Child-Pugh class	
A	177 (74.1)
B	57 (23.8)
C	5 (2.1)
BCLC stage	
0	28 (11.7)
A	46 (19.3)
B	42 (17.6)
C	121 (50.6)
D	2 (0.8)
Tumor size (cm)	7.6 ± 5.7
Tumor number	
Single	113 (47.3)
Multiple	126 (52.7)
PVT	
Presence	98 (41)
Absence	141(59)
Metastasis	
Presence	74 (31)
Absence	165 (69)

Data are expressed as mean ± standard deviation or median (interquartile range).

Data are presented as the n (%) for categorical variable, unless otherwise indicated.

AST, aspartate transaminase; ALT, alanine aminotransferase;TB, total bilirubin; AFP, α-fetoprotein; BCLC, barcelona clinic liver cancer; PVT, portal vein thrombosis.

## Discussion

Metastasis is incurable without early diagnosis tools. Therefore, efficient biomarkers for metastasis that fill gaps of in-depth knowledge should be developed. To this end, we first employed miRNA PCR array to screen serum miRNAs predictive of extrahepatic metastasis. Among several candidate miRNAs, exosomal miR-125b showing the strongest association with metastasis was subjected to more extensive investigations. Based on a series of *in vitro* tests and clinical data, it was found that exosome-mediated miR-125b had significant anti-metastatic properties in HCC. Specifically, the transfer of miR-125b by exosomes inhibited migration and invasion abilities of recipient HCC cells. Exosomal miR-125b also interfered with TGF-β1-induced EMT by suppressing SMAD2 protein expression. Furthermore, a significant downregulation of exosomal miR-125b was detected in a large set of patients with metastasis. More importantly, anti-metastatic effects of exosomal miR-125b were further confirmed by a decrease in its levels at the time of metastasis in patients with serial measurements. These findings indicate the utility of exosomal miR-125b for early diagnosis of extrahepatic metastasis and provide insights into its novel exosome-based therapeutic strategy for inhibiting metastasis in HCC patients.

It is noteworthy that exosomal miRNAs, unlike intracellular miRNAs, modulate cellular processes within recipient cells by indirectly cell-to-cell signaling to distant cells (2). Our observation of cellular internalization and expression of Cy3-labeled miR-125b into recipient cells supports the active role of exosomal miR-125b in cell-to-cell communication (**Figures 1E, F**). It has been reported that miR-125b plays dual roles as an oncogene and a tumor suppressor (13). Oncogenic miR-125b reportedly accelerated cellular proliferation, drug resistance, and migration by controlling target genes in colon, lung, and pancreatic cancers. However, in HCC, miR-125b inhibited these functions by targeting Bcl2, PIGF, LIN28B, and Mcl-1 (13). We found that exosome-mediated delivery of miR-125b effectively mitigated the metastatic potential of recipient HCC cells (**Figures 2A, B**). This finding extends the tumor-suppressive function of cellular miR-125b to the setting of metastasis suppression by exosomal miR-125b and indicates the fundamental role of miR-125b as a key regulator of HCC metastasis. Currently, the role of exosome-mediated signaling in cancer metastasis is highly emerging (7, 22). Tumor cell-derived exosomes can elicit paracrine signaling, whereas exosome-delivered miRNAs mostly target metastasis-related pathways, thereby contributing to the spread of tumors (5, 8, 9). In this regard, exosome-delivered miRNAs, as shown in our results, likely have promising future implications as diagnostic and therapeutic tools for cancer metastasis.

EMT is a key driver that confers metastatic properties on cancer cells by promoting mobility and invasion (23). Among EMT-related markers, activated SMAD2 represents a critical molecule that can accelerate cancer metastasis. SMAD-dependent TGF-β signaling pathways are potent inducers of EMT (21). Therefore, targeting SMAD2 represents one of effective strategies against metastasis. In our study, SMAD2 protein expression was significantly downregulated following exosomal miR-125b transfer in highly metastatic recipient cells (**Figures 3B, C**). Furthermore, when low metastatic Huh7 cells were treated with TGF-β1, exosome-delivered miR-125b also drastically abolished TGF-β1-induced EMT in recipient cell (**Figures 4C–E**). To the best of our knowledge, this is the first study to show the crucial role of exosome-mediated transfer of miR-125b in EMT regulation within recipient cells treated with TGF-β1. Altogether, these results indicate that exosome-delivered miR-125b can repress EMT by inhibiting TGF-β1/SMAD signaling. Thus, it has implications for potential anti-metastatic strategy.

MMPs also play essential roles in metastasis by destroying extracellular matrix (18). MMP-2 and MMP-9 mRNA expression levels have been reported to be upregulated in HCC patients with metastasis (24). Although a number of miRNAs have been implicated in the regulation of MMPs (25), the effects of exosome-associated miRNAs on MMP functions in HCC have not been reported. Through exosome-mediated transfer of miR-125b, we found that MMP-2, MMP-9, and MMP-14 mRNA expression levels were markedly decreased in recipient cells (**Figure 2C**). As MMP-2 was also a direct target of miR-125b (26), the results of the present study indicate powerful anti-metastatic functions of exosome-mediated miR-125b by targeting the two major pathways for metastasis including MMP and EMT process.

It could be argued that observed trends of upregulating exosomal miR-125b with tumor stage progression partly contradict a prior study showing that exosomal miR-125b was downregulated in patients with HCC than in non-HCC patients (27). Such discrepancy might be because of the increased total number of exosomes in tumor cells compared with normal cells and the use of different methods between studies (28, 29). Such paradoxical findings indicate the complexity of exosomal miR-125b in clinical evaluation depending on tumor status. The mechanism of exosome packaging and secretion of miRNAs remains incompletely understood. In addition, our study tested only one donor cell-derived exosome and lacked evaluation of exosomes derived from non-hepatocyte liver cells, such as Kupffer cells, fibroblasts, and stellate cells. Given that exosomes can be secreted by various liver cells besides tumor cells (30), the complex exosome circuitry within the tumor microenvironment could be better evaluated in future studies employing human liver 3D geometrical and functional models.

Among multiple candidate exosome biomarkers developed from basic research, only a few can progress to clinical applications largely because of the lack of verification involving sufficient numbers of well-described patient populations. In this regard, out study has strengths including the recruitment of a large number of patients and serial measurements before and after metastasis for biomarker verification, as well as comprehensive description of exosome-mediated cell-to-cell cargo transfer and its molecular regulation involving metastasis.

In conclusion, this study reveals that tumor-derived, exosome-mediated miR-125b possesses anti-metastatic properties by targeting SMAD2, as well as by inhibiting MMPs and TGF-β1/SMAD signaling pathway in EMT *via* intercellular communication (**Figure 5E**). It also serves as a useful predictor of early metastasis in HCC. These findings highlight that circulating exosomal miR-125b has promising non-invasive diagnostic and therapeutic implications for extrahepatic metastasis of HCC.

## Data Availability Statement

The datasets presented in this study can be found in online repositories. The names of the repository/repositories and accession number(s) can be found in the article/**Supplementary Material**.

## Ethics Statement

This study was approved by the Ethics Committee of The Catholic University of Korea. Informed written consent was obtained from all patients (IRB approval number KC17TESI0664). Written informed consent to participate in this study was provided by the participants’ legal guardian/next of kin. Written informed consent was obtained from the individual(s) for the publication of any potentially identifiable images or data included in this article.

## Author Contributions

Study concept and design: JJ. Acquisition of data: HN, PS, SB, JC, and SY. Analysis and interpretation of data: WH, HK, and JJ. Experiment: HK, NP, JK, and WH. Drafting of the manuscript: HK. Study supervision: JJ. All authors contributed to the article and approved the submitted version.

## Funding

This work was supported by Basic Science Research Program through the National Research Foundation of Korea (NRF) funded by the Ministry of Science, ICT & Future Planning [grant number NRF-2019R1A2C1009439]; the Korea Health Technology R&D Project through the Korea Health Industry Development Institute (KHIDI); and the Ministry of Health & Welfare, Republic of Korea [grant number HI16C2011].

## Conflict of Interest

The authors declare that the research was conducted in the absence of any commercial or financial relationships that could be construed as a potential conflict of interest.

## Publisher’s Note

All claims expressed in this article are solely those of the authors and do not necessarily represent those of their affiliated organizations, or those of the publisher, the editors and the reviewers. Any product that may be evaluated in this article, or claim that may be made by its manufacturer, is not guaranteed or endorsed by the publisher.

## Abbreviations

AFP, α-fetoprotein; ALT, alanine aminotransferase; AST, aspartate transaminase; BCLC, barcelona clinic liver cancer; CHB, chronic hepatitis B; CM, conditioned media; EMT, epithelial to mesenchymal transition; HC, healthy control; HCC, hepatocellular carcinoma; LC, liver cirrhosis; miR, micro RNA; MMP, matrix metalloproteinase; mRNA, messenger RNA; NC, negative control; NTA, nanoparticle tracking analysis; PCR, polymerase chain reaction; PVT, portal vein thrombosis; qRT-PCR, quantitative real-time polymerase chain reaction; RT-PCR, reverse transcription polymerase chain reaction; SEM, standard error of the mean; SFM, serum-free media; SMAD, small mothers against decapentaplegic; TB, total bilirubin; TEI, total exosome isolation kit; TEM, transmission electron microscopy; TGF-β, transforming growth factor beta; UTR, untranslated region.
