# Supplementary material for: Exosomal miR-125b Exerts Anti-Metastatic Properties and Predicts Early Metastasis of Hepatocellular Carcinoma
Source: Front Oncol. 2021 Jul 27;11:637247. doi: 10.3389/fonc.2021.637247 (PMC8354570; doi:10.3389/fonc.2021.637247)
Supplement: Supplementary file 1 [file DataSheet_1.docx]

Supplementary Material

# Supplementary Data

**Supplementary materials and methods**

**Cell lines and cultures**

Human HCC cell lines (Huh7, SK-HEP-1, and SNU449) were obtained from Dr. Yoon (Seoul St. Mary’s Hospital, South Korea). Huh7 and SK-HEP-1 cells were maintained in Dulbecco’s modified Eagle medium (DMEM; Hyclone, Logan, UT, USA) supplemented with 10% fetal bovine serum (FBS), 1% antibiotic-anti mycotic, and 1% HEPES. SNU449 cells were maintained in minimum essential medium (MEM; Gibco, Carlsbad, CA, USA) supplemented with 10% FBS, 1% antibiotic-anti mycotic, 1% HEPES, 1% MEM non-essential amino acids solution, and 1% Sodium pyruvate. All cells were cultured at 37°C in a humidified chamber with 95% air and 5% CO_2_.

**Western blot assay**

Cells and exosomes were lysed with RIPA lysis buffer (Thermo Fisher Scientific, Rochester, NY, USA) or PRO-PREP™ Protein Extraction Solution (Intron Biotechnology, Seoul, Korea) containing a protease inhibitor cocktail (Sigma-Aldrich, St Louis, MO, USA), cocktail 2, and cocktail 3 (Sigma-Aldrich). Equal concentration of protein was subjected to SDS-PAGE and transferred to a PVDF membrane. The membrane was incubated with primary antibody for overnight at 4°C. Protein bands were detected using Clarity Western ECL substrate (Bio-Rad, Hercules, CA, USA). The following primary antibodies were used: CD63 (sc-15363, Santa Cruz Biotechnology, CA, USA), HSP70 (C92F3A-5, Enzo life sciences, Farmingdale, NY, USA), SMAD2 (5339, Cell signaling Technology, Danvers, MA, USA), SMAD2/3 (8685S, Cell signaling Technology), p-SMAD2/3 (8828S, Cell signaling Technology), E-cadherin (3295S, Cell signaling Technology), N-cadherin (610921, BD Biosciences, San Jose, CA, USA), MMP2 (sc-10736, Santa Cruz Biotechnology), STAT3 (ab76315, Abcam Technology, Cambridge, MA, USA), SOD2 (ADI-SOD-110-D, Enzo life science), and β-actin (A5441, Sigma-Aldrich). All experiments were performed in duplicate or triplicate.

**Reverse transcription and quantitative real-time polymerase chain reaction (qRT-PCR)**

Total RNA was extracted from HCC cells and exosomes using Qiazol reagent (Qiagen, Germany) and miRNeasy mini kit (Invitrogen), respectively. cDNA was synthesized using Taqman microRNA reverse transcription kit or High-capacity cDNA reverse transcription kit (Applied Biosystems, Foster City, CA,USA). Expression levels of miRNAs and EMT markers were measured by quantitative real-time PCR (qRT-PCR) using a Taqman MicroRNA assay kit (4427975; Applied Biosystems) and a Taqman 2x master mix on an ABI ViiA7 Real-time PCR system. Relative expression levels were normalized to the expression levels of hsa-miR-16, U6 snRNA, or GAPDH. The following Taqman probes were used: E-cadherin (Hs01023895), N-cadherin (Hs00983056), Vimentin (Hs00958111), hsa-miR-16 (000391), hsa-miR-100-5p (000437), hsa-miR-125b-5p (000449), hsa-miR-130a-3p (000454), hsa-miR-3180-3p (245615_mat), hsa-miR-320a (002277), hsa-miR-26b-5p (000407), hsa-miR-145-5p (002278), U6 snRNA (001973), and GAPDH (Hs03929097). The relative expression levels were determined according to the 2^-∆∆CT^ method (1).

**Reference**

1. Rao X, Huang X, Zhou Z, Lin X. An improvement of the 2ˆ (–delta delta CT) method for quantitative real-time polymerase chain reaction data analysis. *Biostat Bioinforma Biomath*. (2013) 3:71.

# Supplementary Tables and Figures

**2.1 Supplementary Table**

**Table S1.** Primer sequences for reverse transcription PCR

| **Target genes** | **Primer** | **Sequences (5’-3’)** | **Annealing temperature (°C)** |
| --- | --- | --- | --- |
| MMP-2 | Forward | CAGCGATGGCTTCCTCTG | 56 |
|  | Reverse | GCTGTCCTTCAGCGTTGC |  |
| MMP-9 | Forward | TCGTCATCCAGTTTGGTGTC | 55 |
|  | Reverse | ATGGGCGTCTCCCTGAAT |  |
| MMP-14 | Forward | CTGTCAGGAATGAGCATCTGAA | 56 |
|  | Reverse | AGGGGTCACTGGAATGCTC |  |
| GAPDH | Forward | GAGTCAACGGATTTGGTCGT | 54 |
|  | Reverse | TTGATTTTTGGAGGGATCTCG |  |

**Table S2.** Funtional annotation of the miR-125b-5p target genes using DAVID.

| Category | Term | [Target](https://david.ncifcrf.gov/chartReport.jsp?d-16544-p=1&d-16544-o=1&annot=59%2C12%2C87%2C88%2C30%2C38%2C46%2C3%2C5%2C55%2C53%2C70%2C79&currentList=1&d-16544-s=5) genes | [P-Value](https://david.ncifcrf.gov/chartReport.jsp?d-16544-p=1&d-16544-o=1&annot=59%2C12%2C87%2C88%2C30%2C38%2C46%2C3%2C5%2C55%2C53%2C70%2C79&currentList=1&d-16544-s=7) |
| --- | --- | --- | --- |
| KEGG_PATHWAY | [TGF-beta signaling pathway](https://david.ncifcrf.gov/kegg.jsp?path=hsa04350$TGF-beta%20signaling%20pathway&termId=550028739&source=kegg) | SMAD2, SMURF1, ACVR2B, CDKN2B, IFNG, PPP2CA, TGFBR1 | 0.004 |
| KEGG_PATHWAY | [Pancreatic cancer](https://david.ncifcrf.gov/kegg.jsp?path=hsa05212$Pancreatic%20cancer&termId=550028862&source=kegg) | E2F2, SMAD2, ERBB2, MAPK10, STAT3, TGFBR1 | 0.006 |
| KEGG_PATHWAY | [Protein processing in endoplasmic reticulum](https://david.ncifcrf.gov/kegg.jsp?path=hsa04141$Protein%20processing%20in%20endoplasmic%20reticulum&termId=550028723&source=kegg) | BAK1, BCL2, SEL1L, YOD1, ATXN3, MAN1B1, MAPK10, MAP2K7, UBE2G1 | 0.010 |
| KEGG_PATHWAY | [Sphingolipid signaling pathway](https://david.ncifcrf.gov/kegg.jsp?path=hsa04071$Sphingolipid%20signaling%20pathway&termId=550028714&source=kegg) | BCL2, GAB2, ACER2, MAPK10, PPP2CA, PPP2R5C, SGPL1 | 0.019 |
| KEGG_PATHWAY | [Neurotrophin signaling pathway](https://david.ncifcrf.gov/kegg.jsp?path=hsa04722$Neurotrophin%20signaling%20pathway&termId=550028773&source=kegg) | BCL2, SH2B3, MAPK10, MAP2K7, MAP3K1, MAP3K3, RPS6KA1 | 0.019 |
| KEGG_PATHWAY | [HIF-1 signaling pathway](https://david.ncifcrf.gov/kegg.jsp?path=hsa04066$HIF-1%20signaling%20pathway&termId=550028711&source=kegg) | BCL2, MKNK2, ERBB2, IFNG, IL6R, STAT3 | 0.027 |
| KEGG_PATHWAY | [Focal adhesion](https://david.ncifcrf.gov/kegg.jsp?path=hsa04510$Focal%20adhesion&termId=550028744&source=kegg) | BCL2, COL4A3, COL11A2, ERBB2, ITGA8, MAPK10, PPP1CA, PPP1R12B, VAV3 | 0.028 |
| KEGG_PATHWAY | [Phosphatidylinositol signaling system](https://david.ncifcrf.gov/kegg.jsp?path=hsa04070$Phosphatidylinositol%20signaling%20system&termId=550028713&source=kegg) | CDS2, IP6K1, IPMK, POOK, PIK3C2B, PIP4K2B | 0.029 |
| KEGG_PATHWAY | [Adipocytokine signaling pathway](https://david.ncifcrf.gov/kegg.jsp?path=hsa04920$Adipocytokine%20signaling%20pathway&termId=550028796&source=kegg) | ACACB, ACSL6, MAPK10, PRKAA2, STAT3 | 0.034 |
| KEGG_PATHWAY | [Insulin signaling pathway](https://david.ncifcrf.gov/kegg.jsp?path=hsa04910$Insulin%20signaling%20pathway&termId=550028786&source=kegg) | MKNK2, ACACB, FLOT2, MAPK10, PRKAA2, PPP1CA, SOCS4 | 0.034 |
| KEGG_PATHWAY | [Insulin resistance](https://david.ncifcrf.gov/kegg.jsp?path=hsa04931$Insulin%20resistance&termId=550028803&source=kegg) | ACACB, MAPK10, PRKAA2, PPP1CA, PRS6KA1, STAT3 | 0.041 |
| KEGG_PATHWAY | [Epstein-Barr virus infection](https://david.ncifcrf.gov/kegg.jsp?path=hsa05169$Epstein-Barr%20virus%20infection&termId=550028853&source=kegg) | BCL2, ENTPD1, MAPK10, MAP2K7, NCOR2, STAT3 | 0.063 |
| KEGG_PATHWAY | [Dopaminergic synapse](https://david.ncifcrf.gov/kegg.jsp?path=hsa04728$Dopaminergic%20synapse&termId=550028779&source=kegg) | MAPK10, KCNJ6, KCNJ9, PPP1CA, PPP2CA, PPP2R5C | 0.075 |
| KEGG_PATHWAY | [mRNA surveillance pathway](https://david.ncifcrf.gov/kegg.jsp?path=hsa03015$mRNA%20surveillance%20pathway&termId=550028686&source=kegg) | SMG5, CPSF6, PPP1CA, PPP2CA, PPP2R5C | 0.076 |
| KEGG_PATHWAY | [Osteoclast differentiation](https://david.ncifcrf.gov/kegg.jsp?path=hsa04380$Osteoclast%20differentiation&termId=550028742&source=kegg) | FOSL2, GAB2, IFNG, MAPK10, MAP2K7, TGFBR1 | 0.081 |
| KEGG_PATHWAY | [Pathways in cancer](https://david.ncifcrf.gov/kegg.jsp?path=hsa05200$Pathways%20in%20cancer&termId=550028854&source=kegg) | BCL2, E2F2, ETS1, ARHGEF1, SMAD2, COL4A3, DVL3, ERBB2, MAPK10, STAT3, TGFBR1 | 0.084 |
| KEGG_PATHWAY | [FoxO signaling pathway](https://david.ncifcrf.gov/kegg.jsp?path=hsa04068$FoxO%20signaling%20pathway&termId=550028712&source=kegg) | SMAD2, CDKN2B, MAPK10, PRKAA2, STAT3, TGFBR1 | 0.087 |
| KEGG_PATHWAY | [Colorectal cancer](https://david.ncifcrf.gov/kegg.jsp?path=hsa05210$Colorectal%20cancer&termId=550028860&source=kegg) | BCL2, SMAD2, MAPK10, TGFBR1 | 0.095 |

## 2.2 Supplementary Figures

**
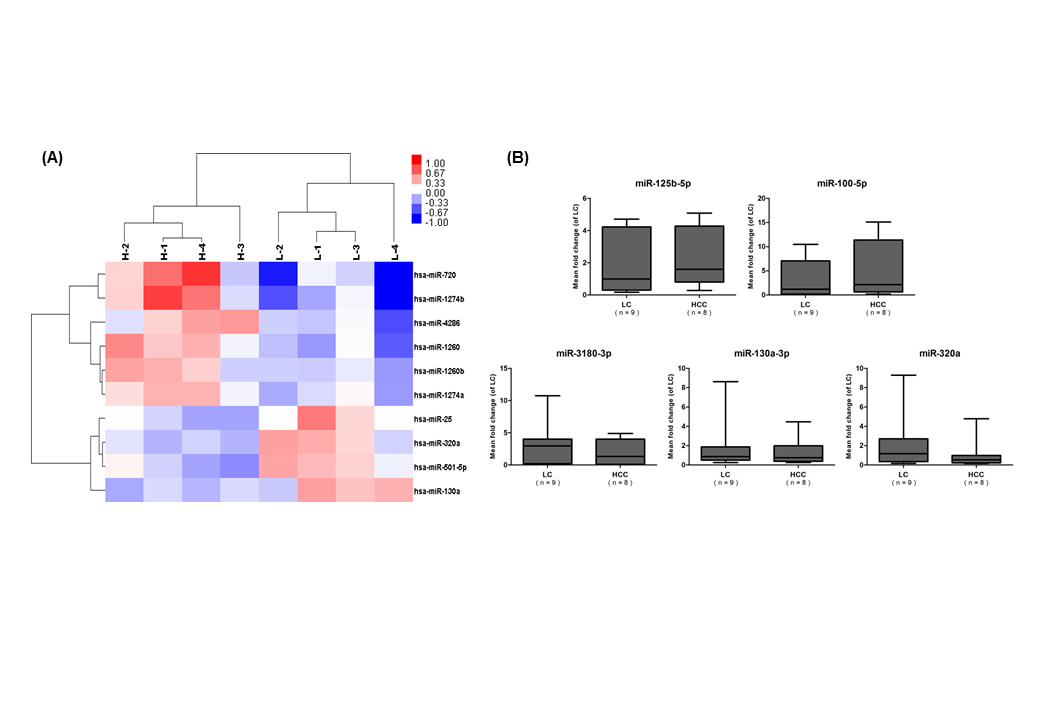
**

**Figure S1.** Screening of extracellular miRNAs in sera of patients. (A) Microarray for non-HCC cirrhotic patients and HCC patients. (B) Expression levels of candidate extracellular miRNAs determined by qRT-PCR.

**
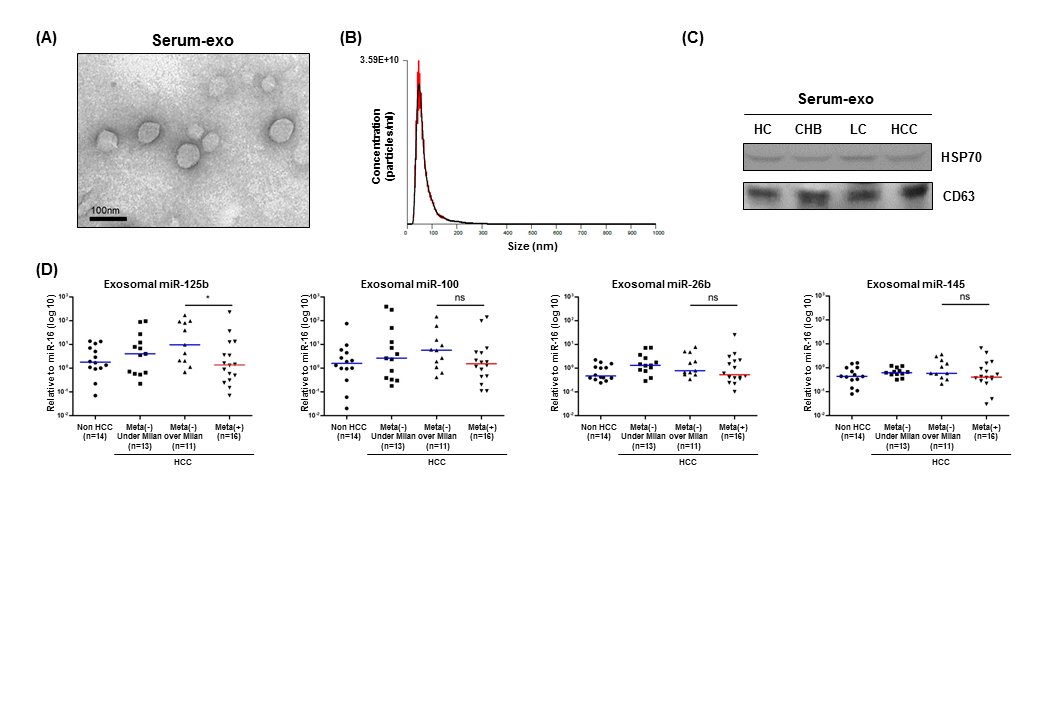
Figure S2.** Exosome characterization and validation of four exosomal miRNAs in sera of HCC patients. Exosomes were isolated from sera of patients. (A) Exosomes morphology by TEM. Images showed that isolated exosomes were round-shaped vesicles. Scale bar, 100 nm. (B) Size distribution and concentration of exosomes were determined by NTA. (C) Exosomal markers detected by Western blot assay. (D) Expression levels of exosomal miRNAs determined by qRT-PCR. miR-16 was used as an endogenous control. Data are presented as median. Mann-Whitney U test was used for data analysis. *, *p* < 0.05; **, *p* < 0.01; ***, *p* < 0.001.

**
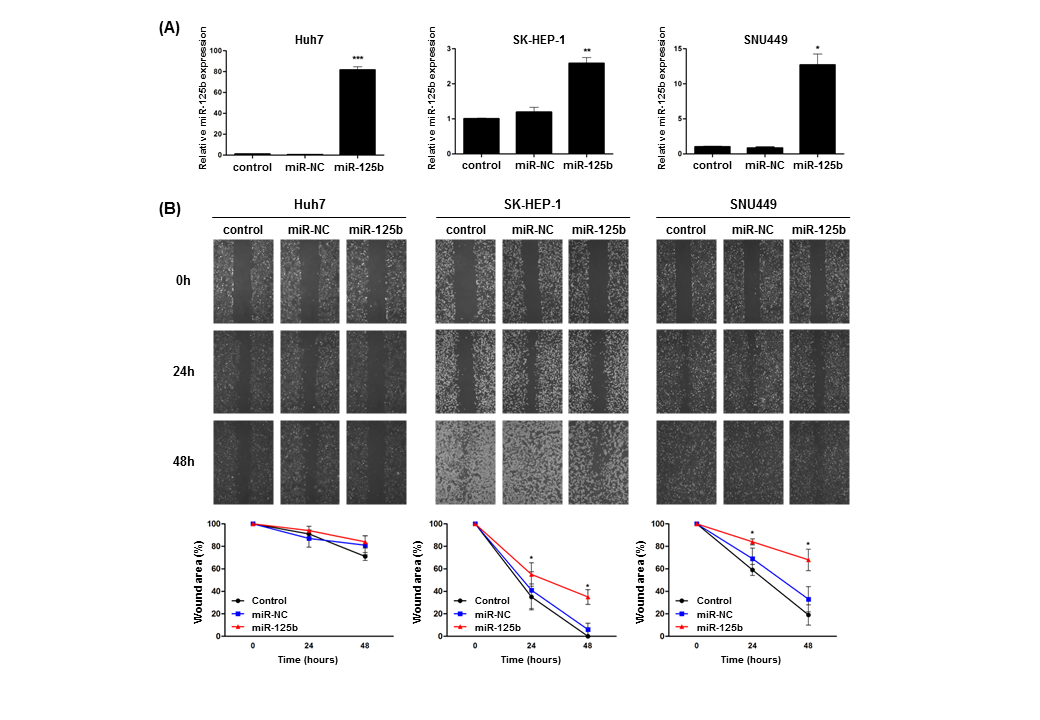
**

**Figure S3.** miR-125b inhibits migration ability of HCC cells. HCC cells were transfected with miR-125b mimic. (A) Transfection efficiency was determined by qRT-PCR. Cell migration ability was assessed by wound healing assay. (B) Percent (%) of wound area was determined compared to that of miR-125b. The difference of wound area was slowly decreased in HCC cells after transfection with miR-125b mimic. Data are presented as mean ± SEM. *, *p* < 0.05; **, *p* < 0.01; ***, *p* < 0.001 vs. miR-NC.

**
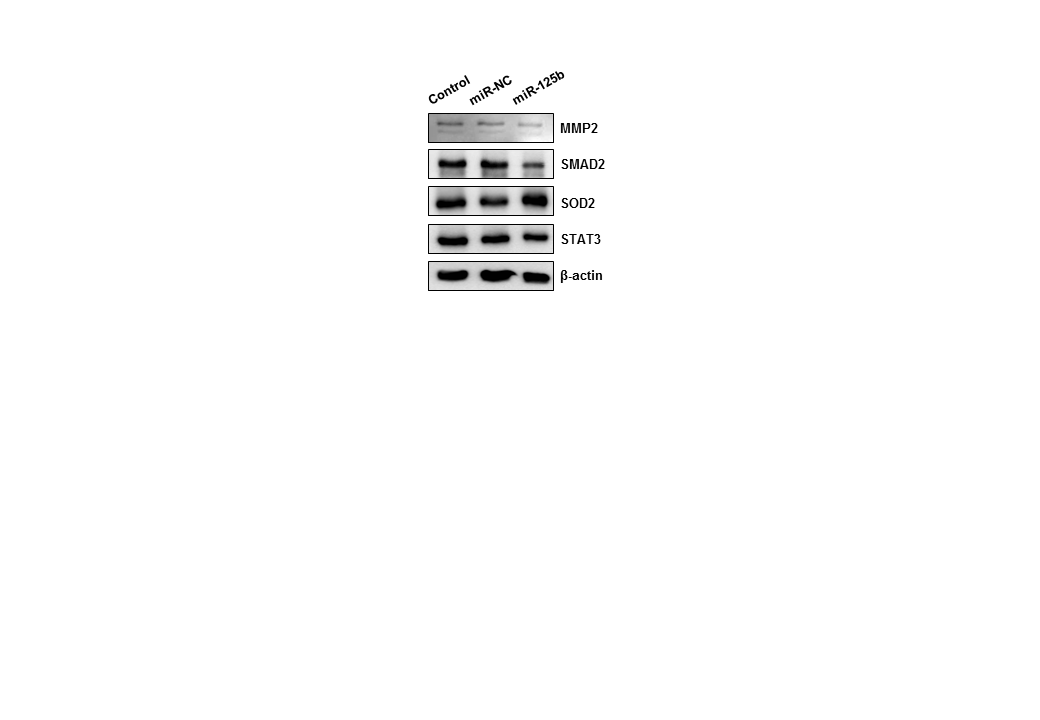
Figure S4.** Candidate target genes of miR-125b were screened by western blot assay. Protein levels of target genes in Huh7 cells transfected with miR-125b were detected by western blot assay.
